# A comprehensive scoring system in correlation with perioperative airway management for neonatal Pierre Robin Sequence

**DOI:** 10.1371/journal.pone.0189052

**Published:** 2017-12-07

**Authors:** Ning Yin, Lei Fang, Xiaohua Shi, Hongqiang Huang, Li Zhang

**Affiliations:** 1 Department of Anesthesiology, Sir Run Run Hospital Affiliated to Nanjing Medical University, Nanjing, China; 2 Department of Anesthesiology, Zhongda Hospital Affiliated to Southeast University, Nanjing, China; 3 School of Medicine, Southeast University, Nanjing, China; 4 Department of Anesthesiology, Nanjing Children’s Hospital Affiliated to Nanjing Medical University, Nanjing, China; Boston Children’s Hospital / Harvard Medical School, UNITED STATES

## Abstract

**Purpose:**

To evaluate a comprehensive scoring system which combines clinical manifestations of Pierre Robin Sequence (PRS) including severity of breathing difficulties, body weight and preoperative Cormack-Lehane grade, for its correlation with perioperative PRS airway management decision.

**Design:**

Forty PRS children were retrospectively recruited after surgery. Specialists examined all subjects and scored for clinical manifestations (1´ - 4´), weight gain (1´- 4´), dyspnea scores (1´- 4´), and Cormack-Lehane grade (1´- 4´). The correlation of the integrated scores and the necessity of endotracheal intubation or laryngeal mask application were analyzed. In addition, the score correlation with postoperative dyspnea and/or low pulse oxygen saturation (SPO_2_) levels after extubation was determined.

**Findings:**

In our study every individual patient had a score from 0´ to 16´, while the higher in the numbers represented higher risk of breathing difficulty. All patients with comprehensive scores <10 points underwent endotracheal intubation successfully. Patients scoring 10–12 points had an intubation success rate of 47%, whereas all patients scored >13 points required a laryngeal mask assisted airway management and were considered to have difficult airways. Dyspnea after extubation and postoperative low SPO_2_ occurred among patients who scored over 10 points.

**Conclusion:**

In PRS patients, preoperative weight gaining status and severity of dyspnea in combination with Cormack-Lehane classification provide a scoring system that could help to optimize airway management decisions such as endotracheal intubation or laryngeal mask airway placement and has the potential to predict postoperative dyspnea or low SPO_2_ levels.

## Introduction

The Pierre Robin Sequence (PRS) was first described in 1923, by a French stomatologist. Infants with PRS are characterized by mandibular hypotrophy (micrognathia) and glossoptosis (abnormal posterior placement of the tongue), which result in serious airway obstruction and feeding difficulties. Other clinical features may also include a soft or high-arched cleft palate, and a typical "bird face" appearance due to the shortened length of the lower jaw [1, 2]. This is a neonatal disease with an occurrence around 1:8500 to 14,000 at births. It is estimated that around 70% of PRS patient with mild airway obstruction could be successfully managed by supine positioning. However, from moderate to severe PRS patients that fail to respond to conservative treatment, additional interventions are necessary. In the most severe cases, a tracheostomy may be ultimately necessary to establish an efficient and permanent airway. Tongue posterior placement, which occurs due to mandibular dysplasia, cleft palate, and large size of the tongue, prevents the infant from effective fetal swallowing [3]. Thus, PRS children often show signs of feeding difficulties, such as extended feeding times, reduced nutritional uptake, and even requirement of feeding by gavage (oral or nasal) [4, 5]. Eventually, PRS children may develop poor nutritional status, inability of gaining weight, and slow growth.

PRS children require anesthesia for a variety of procedures, including tongue-lip adhesion, distraction osteogenesis (DO) of the mandible, and even tracheostomy. These therapies will help to alleviate the breath difficulty and delayed neurodevelopment caused by hypoxia from upper respiratory tract obstructions [6, 7]. However, the clinical features of PRS challenge both anesthesiologists and the surgeons due to the high risk of airway obstruction and difficult intubation. The deformations may cause intraoperative and postoperative complications due to PRS patients’ high risk of upper airway obstruction and intubation/ventilation failure, as well as other preoperative comorbidities such as pneumonia and/or heart failure. In order to reduce the risk of such complications it is important to clearly define the severity of airway obstruction and take appropriate decisions on either mask ventilation, mask ventilation in assistant with oral pharyngeal airways, nasal pharyngeal airways, or laryngeal airways [8]. However, the degree of micrognathia is not always correlated with the degree of airway compromise, as well as laryngoscopy grades, for instance Cormack-Lehane classification alone [9]. Therefore we performed a retrospective investigation aiming to develop a preoperative comprehensive scoring system, which facilitates airway evaluation of PRS patients and may be used to imply specific airway management strategies. Additionally, the comprehensive scoring system was aimed to help shortening intubation time and reducing the incidence of perioperative hypoxia.

## Materials and methods

The study described in this manuscript has been performed according to the Declaration of Helsinki. And the procedures performed in our study was approved by the Independent Ethics Committee for Clinical Research of Zhongda Hospital, Affiliated to Southeast University (Nanjing, China).

### 1.1 Demographic information of the patients

Forty PRS infants (23 males and 17 females) were recruited and analyzed after they underwent surgical treatment between 2007 and 2012 in Nanjing Children´s Hospital. The cohort included 9 premature births and 31 full-term births (average body weight 2.68±1.31 kg). The patients’ ages at surgery time were younger than 30 days after birth in general. Three children were operated at less than 20 days, 13 children were operated between 20–25 days, and 24 children between 26–30 days. In all cases, the American Society of Anesthesiologists (ASA) classification ranged category I—III. The preoperative comorbidities included dyspnea and feeding difficulties are summarized in Table 1.

**Table 1:** Patient distribution on breath and feeding difficulties (preoperative).

		Cases (%)
Upon breath	Chest Retraction Signs*	19 (47%)
	Polypnea	6 (15%)
The usual position	Side	21 (52%)
	Prone	6 (15%)
	Head low	3 (7%)
Feeding difficulties	Feeding time extended	23 (65%)
	Vomiting	12 (30%)
	Nasogastric feeding	3 (7%)

*Chest Retraction Signs: intercostal retractions, supraclavicular retractions, and/or suprasternal retractions

### 1.2 Assessment criteria

All assessment criteria for the scoring system are summarized in Table 2

**Table 2:** Comprehensive scoring system.

Assessment Categories	Scoring Criteria	Score
Clinical Assessment	Small micromaxillary deformity or bird face with jaw retraction	1
	Cleft palate or high palatine arches	1
	In need of oxygenation/ventilation due to tongue posterior placement	1
	Feeding difficulties	1
Body Weight and Growth Assessment	Weight gain 10–20 g/d	1
	Weight gain 5–10 g/d	2
	Weight gain <5 g/d	3
	Surgical intervention required (i.e., weighing less than the birth weight)	4
Dyspnea scores Assessment	Inspiratory dyspnea (three depressions sign) with SPO_2_ level of 92%-96%	1
	Inspiratory dyspnea with SPO_2_ level of 88%-92%, SPO_2_ difference < 5% changing position	2
	Inspiratory dyspnea with SPO_2_ level of <88, SPO_2_ difference > 5% changing position	3
	Continuous positive airway pressure (CPAP)	4
Cormack-Lehane Classification	Visibility of the majority of the glottis	1
	Visibility only a glimpse of the posterior glottis joint	2
	Visibility only a glimpse of the epiglottis, without the appearance of the glottis	3
	Unable to see any anatomical portion of the throat	4
Final Score		

#### 1.2.1 Clinical assessment

According to the clinical features of PRS, one point was accredited for each of the following factors: (1) small micromaxillary deformity or bird face with jaw retraction, (2) cleft palate or high palatine arches, (3) In need of oxygenation/ventilation due to tongue posterior placement, and (4) feeding difficulties.

#### 1.2.2 Body weight and growth assessment

The first week after birth, healthy neonates experienced a temporary weight loss, the so-called physiological weight loss. However, 7–10 days after birth their weight will steadily be restored to the birth weight. In contrast, pathological weight loss is the condition when the weight loss is > 10% of birth weight or could not return to the birth weight by the 10^th^ day. Healthy full-term infants gain up to 1–1.7 kg during the first months after birth [^10^], and the projected neonatal weight gain is >20 g/day. Therefore, for PRS patients with a weight gain between 10–20 g/day 1 point was assigned; for weight gain between 5–10 g/day 2 points were assigned, and for weight gain < 5 g/day 3 points were assigned. Four points were assigned when patients even lost weight compared to their birth weight. It is worth to mention that body weight and growth evaluation was determined at the time when patients were admitted to the hospital before any other treatment or nutritional management.

#### 1.2.3 Dyspnea scores assessment

First, each child was placed in a standard lateral position with pulse oxygen saturation (SPO_2_) monitoring and then slowly moved into the supine position. The SPO_2_ level was recorded in both positions. The scoring method assigned: 1 point for the presence of inspiratory dyspnea (Chest Retraction Signs: intercostal retractions, supraclavicular retractions, and/or suprasternal retractions) with SPO_2_ levels between 92%-96%; 2 points for breathing difficulties with SPO_2_ level between 88%-92% and a SPO_2_ difference between the lateral and the supine positions < 5%; 3 points for inspiratory dyspnea and SPO_2_ level < 88% and a SPO_2_ difference > 5% upon position change; and finally 4 points for continuous positive airway pressure (CPAP).

#### 1.2.4 Cormack-Lehane classification

The Cormack-Lehane classification was determined by using the follow rubric and a laryngoscope: visibility of the majority of the glottis (level 1 = 1 point), only a glimpse of the posterior glottis joint (level 2 = 2 points), only a glimpse of the epiglottis, without the appearance of the glottis (level 3 = 3 points), or unable to see any anatomical portion of the throat (level 4 = 4 points) [11].

### 1.3 Anesthesias and monitoring

All patients were fasted for 4 hours without any premedication before surgery. A multi-parameter monitor was connected to measure the heart rate (HR), blood pressure (BP), and SPO_2_, and a standard preparation of atropine (0.1 mg/ml), epinephrine (5 μg/ml), and aminophylline (5 mg/ml) was ready to use. However, they were only administered when necessary: atropine is used if the infant has decreased heart rate caused by hypoxia or to reduce excessive secretion of saliva; epinephrine is used as a resuscitation drug to counter sudden heart arrest; and aminophylline is prepared to release bronchospasm. Sevoflurane (decreasing from 5% to 1%) was inhaled through a mask, with an oxygen flow at 5 L/min. Slow intravenous administration of 2 mg/kg ketamine were given intravenously to maintain spontaneous respiration until the eyelash reflex disappeared and the HR together with BP decreased 20% as compared to baseline. An airway assessment was performed using the Cormack-Lehane laryngoscope classification. Endotracheal intubation or laryngeal mask placement were performed by trained anesthesiologists (Miller 0, 1, laryngeal mask, Yuyue Medical Co., Shanghai, Jiangsu Province, China) under the assistance of self-made copper intubation stylet, and interval sevoflurane inhalation was maintained to sustain anesthesia.

Any one of the following criteria was considered as a failed intubation: more than 3 intubation attempts, total intubation time >15 minutes, and/or decreased heart rate (Lower than 70-80/min). If there were any intubation difficulties that could not be overcome, intubation was diverted into a laryngeal mask placement as the alternative option, and intraoperative 1–2% sevoflurane anesthesia was administered. Chest auscultation and end tidal carbon dioxide (PETCO_2_) monitoring were applied to judge the successful tracheal intubation or laryngeal mask insertion. After successful intubation or laryngeal mask placement, the anesthesia apparatus was supplemented with mechanical ventilation that was adjusted to maintain the PETCO_2_ at 35–40 mmHg.

In addition to the score evaluation, blood pressure, heart rate, SPO_2_ level, intubation time and intubation attempts were also recorded.

## Results

The scores according to all PRS patients’ clinical manifestations, growth retardations, severities of breathing difficulty, and Cormack-Lehane classification are summarized in Table 3.

**Table 3:** Patient distribution based on comprehensive assessments (cases %).

	Score	1	2	3	4
Evaluations					
Clinical Assessment		0	5 (12.5%)	13 (32.5%)	22 (55%)
Body Weight and Growth Assessments		2 (5%)	13 (32.5%)	19 (47.5%)	6 (15%)
Dyspnea Assessment		11 (27.5%)	18 (45%)	6 (15%)	5 (12.5%)
Cormack-Lehane classification		1 (2.5%)	9 (22.5%)	22 (55%)	8 (20%)

**Fig 1:**
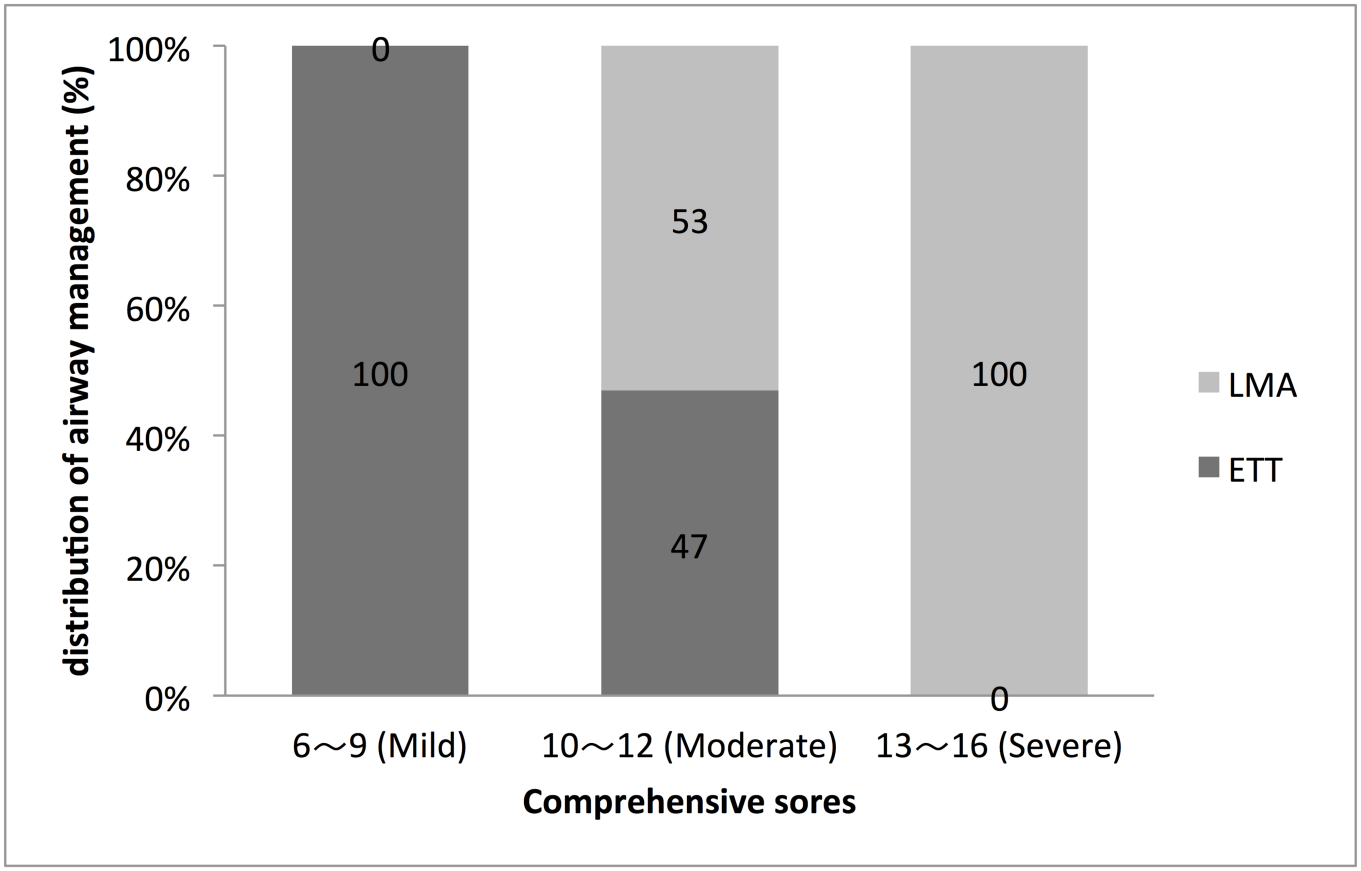
The relationship between comprehensive scores and airway management. (LMA: Laryngeal Mask, ETT: Endotracheal Intubation).

Fig 1 shows that all patients with comprehensive scores between 6 and 9 had been successfully intubated, whereas in patients with scores between 10–12 the intubations were successful in only 47%, and none of the patients with a score > 13 could be intubated. For patients who could not be intubated successfully, a laryngeal mask airway implant was chosen to manage all difficult airways.

Intubation and extubation was achieved in 12 out of 17 cases (Table 4). The remaining 5 patients were treated with continued tracheal catheters and mechanical ventilation in the ICU, because of postoperative pulmonary effusion or heart failure. All 23 patients in the laryngeal mask group were successfully extubated. However, 6 patients experienced serious airway inspiratory dyspnea after the laryngeal mask was removed, and 6 patients developed SPO_2_ levels <95%. Two of these patients had tongue traction suspension, whereas four were treated with nasopharyngeal catheters [reconstruction of the inner diameter (ID) 3.5 catheter, with a 6–8 cm insertion depth. Three days after surgery, the four patients with nasopharyngeal catheters showed a significant increase of the SPO_2_ level (>95%) and an extension was applied to the traction. After the mandibular length reached > 3 mm the nasopharyngeal catheter was removed. Importantly, in our comprehensive scoring system all patients with either postoperative dyspnea or low SPO_2_ levels after extubation / removing the laryngeal mask were scored >10 points, suggesting that the system is predictive for postoperative complications.

**Table 4:** Postoperative airway commodities in 40 PRS patients.

Postoperative commodities	Cases (40)	Laryngeal mask	Tracheal intubation	Correlated Comprehensive score
Difficult decannulation or breathing difficulties	11 (40)	6 (23)	5 (17)	10~12
SPO_2_ <95% after extubation	6 (40)	6 (23)	0 (17)	13~16

## Discussion

In this retrospective study we evaluated preoperative clinical symptoms in 40 neonatal PRS children in a comprehensive scoring system, which aimed to predict the optimized strategy for perioperative airway management and postoperative complications. Based on our score system (1´-16´), the relative difficulty of endotracheal intubation could be predicted in all PRS patients with scores > 13 points, hence consequently a laryngeal airway mask could be applied. Furthermore, the incidence of dyspnea after extubation was significantly increased when the total score was >10.

Airway management during distraction osteotomy is one of the main challenges to anesthesiologists in PRS children. Since the exposure of the glottis is quite difficult in micrognathia patients, consequently the risk of intubation failure is increased and results in prolonged intubation time, repeated trials of intubation and failures. These events often cause severe hypoxia and critical complications. Additionally, the pharyngeal cavity may accumulate bloody secretion due to throat soft tissue trauma, which blurs the laryngoscope visual field and the laryngeal traumatic edema rate may be increased as well. Therefore, it is essential to choose appropriate airway management strategies to ensure airway control within a limited time frame. The usage of laryngeal masks is one of the suitable alternative methods for children with difficult endotracheal intubation as it avoids vocal cord intervention and repeated glottal intubation, thus prevents the occurrence of stenosis. However, this procedure brings several shortcomings as compared to endotracheal intubation. PRS children with mandibular distraction osteogenesis need to undergo bilateral operation, thus the intraoperative side head position must be altered from one side to another. Torsion or displacement of the laryngeal mask can easily occur during repositioning, and even result in severe asphyxia when the top of the cuff slides into the throat. Besides, laryngeal airway mask make the operational space even more limited during the surgery. Therefore, a supplementary endotracheal tube was recommended being inserted through the laryngeal mask for safety consideration by others [12–15].

One previous investigation categorized PRS patients into three groups based on the severity of their breathing and feeding symptoms, which correlated with patient mortality [16]. In our cohort study, preoperative clinical manifestations (difficulties of breathing, body weight factors and Cormack-Lehane grading scores) were collectively evaluated and quantified in a comprehensive scoring system which gave us the tool to assess the patient´s intubation risk. Here we intend to address one issue regarding Cormack-Lehane classification. It might raise confusion as the grading result might differ, which depends critically on the device used to perform laryngoscopy such as type of blade (Miller), approach (classic versus retromolar) or whether indirect laryngoscopy is used. The reason for us to included Cormak-Lehane grade in this study is that this is one direct judgment for anesthesiologist to obtain right before airway establishment, beside any other indirect assessment in our scoring system. In order to reach a certain level of standard result and fare contribution to the grading, laryngoscopies by Miller blades are the universal equipment that used in our institute for this study.

The selection of either a tracheal intubation or laryngeal mask reliability correlated with our score system. Our data strongly supported the hypothesis that the comprehensive scoring system predicts the difficulty of endotracheal intubation and can be applied to guide appropriate airway management for PRS children. Ideally, as one retrospective study on specific preoperative assessment, it would be more interesting and meaningful if there were any other conventional assessment to make a parallel comparison. However, considering PRS patients are mostly neonatal or infants, many standard evaluation methods are not practicable for them. That´s the reason we didn’t apply any conventional assessment to make an equal comparison to our result. As a matter of fact, we want to propose this scoring method described in our manuscript to be further utilized and judged in future studies, which will help to optimize our notion and make contributions to the PRS airway management practice. Additionally, this preoperative scoring system may predict the incidence of postoperative airway obstruction difficulties. In our cases, mandibular distractors should be activated from the first postoperative day and should be provided 3 times/day and at 0.4 mm/time for about 2 weeks or until the desired length of extension is reached. However, there are still risks for a respiratory tract obstruction occurrence during this time. Under this condition, tongue traction suspension or nasopharyngeal catheterization could effectively dissolve the airway obstruction after surgery. Applying with our scoring system, these difficulties occurred in 6 patients who were scored 13–16 points.

In conclusion, the comprehensive scoring system, which combines different clinical manifestations of PRS, strongly correlated with PRS patient airway management strategies. Within the retrospective scale, this scoring system predicted the staging of difficult airways and helped anesthesiologists taking sufficient preparations and judgment on appropriate airway management strategies perioperative. This proposed scoring system also predicted the occurrence of dyspnea or SPO_2_ decline after tracheal extubation. Ideally, this method will help the anesthesiologists to make quick judgment on airway management procedure selection, and save the unnecessary effort on intubation failure, shorten the airway disturbing time and reduce the related perioperative risk. One shortcoming in this work is its retrospective structure, and it is necessary to use prospective study design to validate our report, with larger scale of patient cohort, more standard evaluation techniques and well-trained anesthetists specified for the study. Lastly, more work and concerns should be laid out on making explanations of how to deal with the paradox between scores and actual practice when encountered.

## Supporting information

S1 Table PRS dataset. (PDF)
