# Supplementary material for: A comprehensive scoring system in correlation with perioperative airway management for neonatal Pierre Robin Sequence
Source: PLoS One. 2017 Dec 7;12(12):e0189052. doi: 10.1371/journal.pone.0189052 (PMC5720744; doi:10.1371/journal.pone.0189052)
Supplement: S1 Table — (PDF) [file pone.0189052.s001.pdf]

S1 Table PRS dataset

Case No.	Gender	Age(day)	ASA	Weight(g)	Weight gain	Dyspnea	Cormack-Lehane grade	Endotracheal intubation	Laryngeal mask	Postoperative dyspnea	Postoperative low SPO_2
**1**	Male	28	I	3020	15.1g		II	y			
**2**	Male	25	I	2900	3.4g		III	y		y	
**3**	Female	27	I	2600	9.2g	y	IV		y		
**4**	Male	30	I	3040	4.7g		II	y			
**5**	Female	21	II	2160	6.3g		III		y		
**6**	Female	18	II	2100	5.1g		III		y		
**7**	Male	21	I	2580	8.3g		II	y			
**8**	Male	25	I	2910	2.8g		III		y		
**9**	Male	26	II	3080	3.6g	y	IV		y	y	y
**10**	Female	29	II	3070	9.7g		II		y		
**11**	Male	22	II	2530	7.5g		II	y		y	
**12**	Female	25	I	2910	2.9g		III	y			
**13**	Female	26	II	3020	4.4g		III		y		
**14**	Male	15	III	1970	3.7g	y	IV		y	y	y
**15**	Female	23	I	2580	4.6g		II	y			
**16**	Male	21	II	2290	7.9g		III		y		
**17**	Male	27	I	3070	6.6g		III		y		
**18**	Male	26	I	3050	2.2g		III	y		y	
**19**	Female	29	I	2860	3.9g		III		y		
**20**	Male	21	II	2020	4.1g		III		y		
**21**	Male	28	I	3060	9.3g		II	y			
**22**	Female	24	I	2460	2.1g		III		y		
**23**	Female	26	I	3100	17.2g		I	y			
**24**	Male	28	I	3010	7.5g		III		y		
**25**	Male	27	I	2920	1.8g		II	y		y	
**26**	Male	29	I	3010	4.6g		III	y			
**27**	Male	17	II	2010	2.7g	y	IV		y	y	y
**28**	Female	21	II	2180	6.3g	y	IV		y	y	y
**29**	Male	28	I	2700	3.9g		III		y		
**30**	Female	24	I	2180	4.7g		III	y			
**31**	Female	26	I	2500	8.1g		III		y		
**32**	Male	28	I	2780	2.7g		III		y		
**33**	Female	30	I	2800	6.5g		III		y		
**34**	Female	27	II	2670	3.3g	y	IV		y	y	y
**35**	Male	21	I	2200	7.1g		II	y			
**36**	Female	26	I	2480	8.2g	y	IV		y	y	y
**37**	Male	28	I	3020	5.6g		III	y			
**38**	Female	29	I	2950	4.6g	y	IV		y		
**39**	Male	22	II	2470	6.8g		III	y			
**40**	Male	30	I	3090	2.5g		III	y		y	
